# Total keratometry for toric intraocular lens calculation: comparison from two swept-source optical coherence tomography biometers

**DOI:** 10.3389/fmed.2023.1276186

**Published:** 2024-01-08

**Authors:** Feiyan Chai, Jack X. Ma, Xiaogang Wang, Jiewei Liu, Lin Jiang, Wanmin Wu, Junhong Li

**Affiliations:** ^1^Shanxi Eye Hospital Affiliated to Shanxi Medical University, Taiyuan, Shanxi, China; ^2^Ruiz Department of Ophthalmology and Visual Science, McGovern Medical School at The University of Texas Health Science Center at Houston, Houston, TX, United States

**Keywords:** total keratometry, total corneal power, prediction error, IOLMaster 700, Anterion

## Abstract

**Purpose:**

To compare the astigmatism prediction accuracy of total keratometry (TK) from the IOLMaster 700 and total corneal power (TCP) from Anterion based on swept-source optical coherence tomography (SS-OCT) technology in toric intraocular lens (toric IOL) calculation.

**Design:**

A retrospective observational study.

**Methods:**

Total corneal astigmatism (TCA) were obtained using IOLMaster 700 and Anterion. Z CALC 2.0 was used to calculate the expected postoperative refractive astigmatism in conjunction with TCA. Prediction errors (PE) in refractive outcomes was analyzed 1 month postoperatively using the vector analysis by the Holladay method, including the mean vector PE magnitude, percentage of cases with vector PE in certain intervals, and the centroid PE.

**Results:**

A total of 56 eyes from 56 patients were enrolled in the study with an insertion of an AT TORBI 709 toric IOL. The difference in mean vector PE of postoperative refractive astigmatism between TK and TCP was not statistically significant (0.48D versus 0.46D, *P* = 0.281). TK and TCP yielded 27.3 and 40.0% of eyes with vector PE ≤ 0.25D, and 58.2 and 63.6% with vector PE ≤ 0.5D (both *P* > 0.05), respectively. TK and TCP resulted in similar ATR centroid PE of 0.10D@35° ± 0.60D and 0.15D@22° ± 0.57D, respectively, and there were no significant differences between x-PE component and y-PE component.

**Conclusion:**

IOLMaster 700 and Anterion provided comparable astigmatic predictability in toric IOL implantation using total keratometry and Z CALC 2.0.

## 1 Introduction

Corneal astigmatism is one of the critical factors leading to poor visual acuity after cataract surgery. Although toric intraocular lens (toric IOL) implantation can dramatically improve the refractive outcome of patients with pre-existing corneal astigmatism (1, 2), unexpected residual astigmatism remains a concern, which can be caused by a variety of factors, such as inaccurate preoperative corneal astigmatism measurement (2), surgical-induced corneal astigmatism (SIA), misalignment of toric IOL (3), and inappropriate way to calculate toric IOL diopter. Among the above factors, posterior corneal astigmatism (PCA) has the greatest influence on astigmatic prediction error (4). Much literature has discussed the contribution of PCA to total corneal astigmatism (TCA) (5, 6). Studies have shown that ignoring PCA may cause estimation errors in TCA, leading to overcorrection of with the rule (WTR) astigmatism and undercorrection of the against the rule (ATR) astigmatism (4, 5, 7). Therefore, when calculating the toric IOL power, considering PCA can improve the accuracy of refractive results (8, 9).

At present, with the development of examination technology, there are several biometers that are now available to acquire ocular anterior segment parameters and measure astigmatism on the front and back surface of the cornea, such as Pentacam (Optikgerate GmbH, Wetzlar, Germany), IOLMaster 700 (Carl Zeiss Meditec, Jena, Germany), and Anterion (Heidelberg Engineering, Heidelberg, Germany), of which the latter two were based on swept-source optical coherence tomography (SS-OCT) technology. Using the Alcon online toric calculator, Choi et al. (10) compared the accuracy of TCA from the IOLMaster 700 and Pentacam when implanting of Acrysof IQ Panoptix toric TFNT IOLs (Alcon Laboratories, Inc., Fort Worth, TX). It was found that favorable refractive outcomes were provided by TCA from the two biometers. Wang and Koch (11) compared the accuracy of astigmatic prediction by Barrett toric calculator using the PCA measured by the IOLMaster 700 and the predicted PCA, respectively, and the results showed that astigmatic prediction error (PE) was significantly improved with the measured PCA. However, to our knowledge, no literature has explored the performance of TCP from Anterion in astigmatic prediction. Therefore, this study aimed to compare the PEs in postoperative refractive astigmatism based on TK with the IOLMaster 700 and TCP with the Anterion.

## 2 Patients and methods

### 2.1 Study population

This retrospective study was reviewed and approved by the institutional review board of Shanxi Eye Hospital, Affiliated to Shanxi Medical University (No. SXYYLL-20210107), which conformed to the Declaration of Helsinki principles. A list of patients who underwent toric IOL implantation at Shanxi Eye Hospital was retrospectively obtained from Jan 2021 to July 2022. The inclusion criteria were senile cataract patients with reliable preoperative measurements and regular corneal astigmatism between 0.75D and 5D. The exclusion criteria were cases with systemic diseases and ophthalmologic diseases that affect postoperative refractive and visual acuity, intraoperative or postoperative complications, keratoconus and contact lens wearing history within 2 weeks before preoperative and postoperative measurement. Patients with IOL misalignment of > 5° by the toriCAM Application (12) and postoperative corrected distance visual acuity (CDVA) of < 20/40 were excluded.

### 2.2 Preoperative examination

A thorough ophthalmic examination was performed preoperatively, including uncorrected visual acuity and best-corrected visual acuity, slit-lamp examination, subjective and objective refraction, tonometry, and adequate fundoscopy after dilatation. In this study, two anterior segment SS-OCT biometers (IOLMaster 700 and Anterion) were used. Reliable measurements (quality check “green”) were obtained. Pentacam checking was performed in order to determine the regularity of astigmatism.

### 2.3 IOL power calculation

For postoperative target refraction, 6 eyes were targeted for myopia between −2.0D and −3.0D, and 50 eyes were aimed to emmetropia. The spherical equivalent (SE) and the cylinder power of IOL were determined using Z CALC 2.0 (Carl Zeiss Meditec AG) based on the TK mode (13), in combination with TK, anterior chamber depth and axial length of the IOLMaster 700. It was assumed that the SIA is 0.3D@120° for all cases according to our previous study.

### 2.4 Surgical technique

Before the surgery, all patients were manually marked the meridian of the incision and alignment axis of the intraocular lens in a sitting position under natural pupil by one experienced surgeon (JWL.), who performed all surgeries under topical anesthesia. Phacoemulsification was carried out with a 2.2 mm clear corneal primary incision, followed by an insertion of an AT TORBI 709 toric IOL (Carl Zeiss Meditec AG, Jena, Germany). After IOL implantation, the reference marker of IOL was aligned to the corneal preoperative marks.

### 2.5 Postoperative examination and calculations

Postoperative evaluation was performed at 1 month after surgery, including visual acuity and manifest refraction. Predicted residual astigmatism with the implanted toric IOL was calculated using two sets of values: (1) TK from the IOLMaster 700, and (2) TCP from the Anterion. PEs were calculated as the difference between the actual refractive astigmatism postoperatively and the predicted residual astigmatism (14). Vector analysis was carried out in all calculations according to the Holladay method (15). The mean vector PE magnitude, percentage of eyes within certain intervals of vector PE, as well as the centroid PE were calculated (16).

According to the steep meridian of TK obtained by the IOLMaster 700, eyes were divided into WTR group (the steep meridian at 67.5° to 112.5°) and ATR group (the steep meridian at 0° to 22.5° or 157.5° to 180°), and then subgroup analysis was performed in the two groups. Oblique astigmatism (the steep meridian at 22.5° to 67.5° or 112.5° to 157.5°) was excluded due to small sample size.

### 2.6 Statistical analysis

With a significance level of 5% and a power of 80%, sample size calculation was performed to detect a prediction error of 0.2 D and standard deviation of PE of approximately 0.5 D showed in a preliminary study. The calculation showed that 52 eyes were required.

The main outcomes were vector PE magnitudes, percentage of eyes with vector PEs in certain intervals, and centroid PE of TK and TCP. The R software^1^ and related functions outlined in the study by Holladay et al. (17) were used for statistical analysis. Univariate normality analysis was performed using Shapiro–Wilk test. The difference in total variance of preoperative corneal and actual postoperative refractive astigmatism for each method were compared. The vector PEs for TK method and TCP method were compared using a bootstrap-t method. McNemar test was carried out to compare the percentage of eyes with vector PEs at various intervals between the two methods. *P*-values were corrected by Holm-Bonferroni method. A bivariate analysis of x-PE component and y-PE component of the centroid value was conducted according to the bootstrap-t method. *P* < 0.05 was considered statistically significant.

## 3 Results

Demographic data of the study population are shown in Table 1. A total of 56 eyes from 56 patients were enrolled in the study.

**TABLE 1:** Demographic data of the study population.

Parameters	Mean ± SD (range)
**Patient**	
Male	27
Female	29
**Eye (*n*)**	
Right	37
Left	19
**Type of astigmatism**	
WTR	20
ATR	36
Axis length (mm)	23.81 ± 1.19 (21.01 to 28.58)
TK total corneal astigmatism magnitude (A, D)	2.18 ± 0.72 (1.13 to 4.48)
TCP total corneal astigmatism magnitude (B, D)	2.24 ± 0.75 (1.12 to 4.57)
IOL SE power (D)	17.75 ± 3.24 (5 to 25)
IOL cylinder power (D)	2.79 ± 0.97 (1.5 to 6)
Postoperative spherical equivalent (D)	−0.19 ± 1.00 (−3.38 to 1.63)
Postoperative cylinder power (D)	0.47 ± 0.42 (0 to 1.50)

SD, standard deviation; WTR, with-the-rule astigmatism; ATR, against-the-rule astigmatism; TK, total keratometry form IOLMaster 700; TCP, total corneal power from Anterion; SE, spherical equivalent; D, diopter.

### 3.1 Double-angle plots and total variances

**FIGURE 1:**
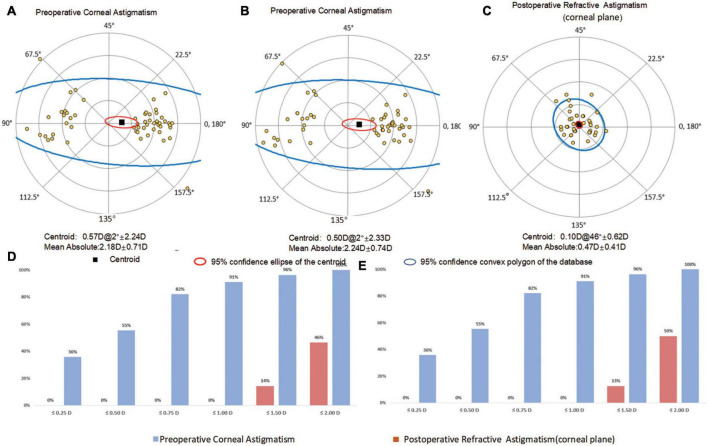
Double-angle plots **(A,B,C)** and a cumulative histogram **(D,E)** of the preoperative corneal and actual postoperative refractive astigmatism for IOLMaster 700 and Anterion. **(A)** TK from IOLMaster 700; **(B)** TCP from Anterion; **(C)** postoperative refractive astigmatism.

Double-angle plots and cumulative histograms of preoperative corneal astigmatism and postoperative refractive astigmatism at corneal plane for TK and TCP were presented in Figure 1. As shown in the figure, there was no statistical difference in the percentages of eyes at various intervals of corneal astigmatism between TK and TCP (± 0.25D to ± 2.0D, with 0.25D interval) before surgery. There were no significant differences in preoperative bivariate variances between TK and TCP (5.1223 versus 5.5019, *P* = 0.081). The postoperative refractive astigmatism values were significantly decreased compared to both preoperative TK and TCP astigmatism (both *P* < 0.001 using Holm correction). Statistical significance was found between preoperative and postoperative bivariate variances (5.1223&5.5019 versus 0.3899, both *P* < 0.001).

### 3.2 Mean vector prediction error

**FIGURE 2:**
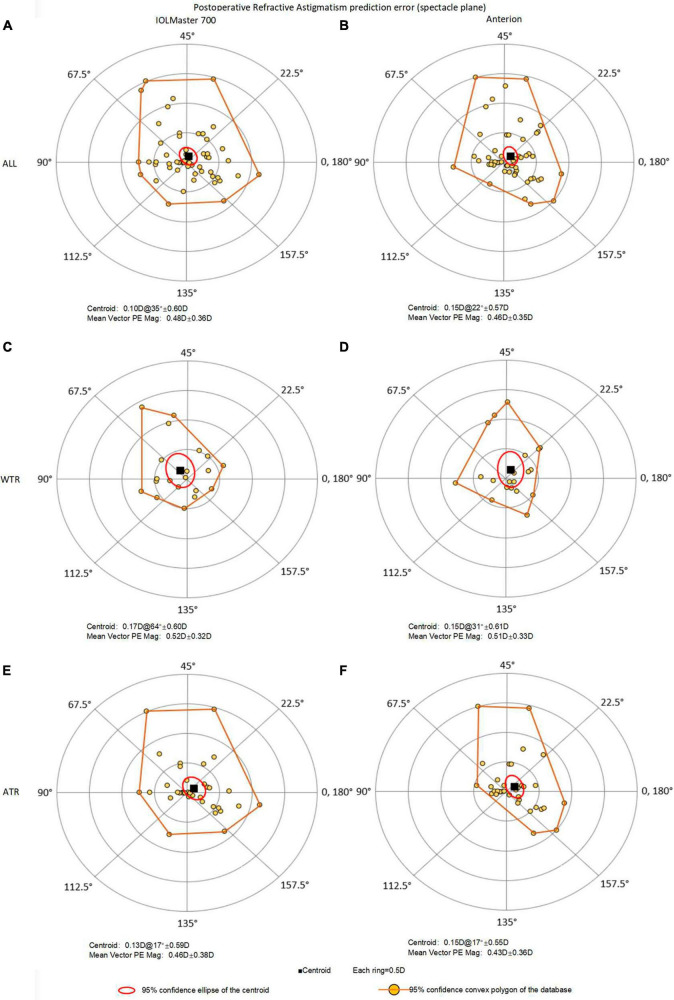
Double-angle plots of astigmatic prediction error with 95% confidence convex polygon using total keratometry (TK) from IOLMaster and total corneal power (TCP) from Anterion for all eyes (top, **A**: IOLMaster; **B**: Anterion), WTR eyes (middle, **C**: IOLMaster; **D**: Anterion) and ATR eyes (bottom, **E**: IOLMaster; **F**: Anterion). ATR, against-the-rule astigmatism; WTR, with-the-rule astigmatism; prediction error = postoperative refractive astigmatism (spectacle plane) - predicted residual astigmatism (spectacle plane).

The mean vector magnitude PEs were 0.48D and 0.46D for TK and TCP, respectively, with no statistically significant difference (*P* = 0.281) (Figure 2). Similarly, no statistical differences were found for percentages of eyes within different intervals of vector PEs (all *P* > 0.05). Compared with TK, the percentages of eyes with TCP increased by 12.7 and 5.4% within the intervals of vector PEs ≤ 0.25D and ≤ 0.5D, respectively (Table 2); however, these improvements were not statistically significant.

**TABLE 2:** The percentage of eyes with vector PE magnitudes below certain intervals and the *P*-value for comparison (Holm corrected) between TK and TCP.

Interval (D)	All (56)			WTR (20)			ATR (36)		
	**TK**	**TCP**	***P*-value**	**TK**	**TCP**	***P*-value**	**TK**	**TCP**	***P*-value**
≤ 0.25	27.3%	40.0%	0.070	15.8%	26.3%	0.617	34.3%	48.6%	0.131
≤ 0.50	58.2%	63.6%	0.505	63.2%	63.2%	1.000	57.1%	65.7%	0.371
≤ 0.75	81.8%	80.0%	1.000	84.2%	78.9%	1.000	80.0%	80.0%	1.000
≤ 1.00	90.9%	90.9%	1.000	89.5%	89.5%	1.000	91.4%	91.4%	1.000
≤ 1.25	94.6%	94.6%	1.000	94.7%	94.7%	1.000	94.3%	94.3%	1.000
≤ 1.50	98.2%	100%	1.000	100%	100%	1.000	97.1%	100%	1.000
≤ 1.75	100%	100%	1.000	100%	100%	1.000	100%	100%	1.000
≤ 2.00	100%	100%	1.000	100%	100%	1.000	100%	100%	1.000

PE, predicted error; TK, total keratometry form IOLMaster 700; TCP, total corneal power from Anterion; D, diopter; WTR, with-the-rule astigmatism; ATR, against-the-rule astigmatism.

Subgroup analysis showed that the vector PEs for TK and TCP were 0.52D and 0.51D in the WTR eyes, and 0.46D and 0.43D in the ATR eyes, respectively. There were no significant differences between TK and TCP in WTR eyes (*P* = 0.593) and ATR eyes (*P* = 0.245).

### 3.3 Centroid prediction error

Figure 2 and Table 3 presented double-angle plots of prediction errors for TK and TCP, with a brownish-yellow convex polygon representing 95% confidence boundary of the data. The centroid values were 0.10D@35° ± 0.60D for TK and 0.15D@22° ± 0.57D for TCP. There were no significant differences in x-PE and y-PE components between the two methods.

**TABLE 3:** x- and y- component vector prediction errors.

Parameter	TK						TCP					
	**All (56)**		**WTR (20)**		**ATR (36)**		**All (56)**		**WTR (20)**		**ATR (36)**	
	**Act-Pre**		**Act-Pre**		**Act-Pre**		**Act-Pre**		**Act-Pre**		**Act-Pre**	
	**x**	**y**	**x**	**y**	**x**	**y**	**x**	**y**	**x**	**y**	**x**	**y**
*N*	56	56	20	20	36	36	56	56	20	20	36	36
Mean (D)	0.03	0.10	−0.10	0.13	0.11	0.07	0.11	0.10	0.07	0.13	0.12	0.08
SD	0.40	0.44	0.35	0.46	0.40	0.43	0.33	0.46	0.33	0.49	0.32	0.43
STD ERR	0.05	0.06	0.08	0.10	0.07	0.07	0.04	0.06	0.07	0.11	0.05	0.07
Median	0	0	−0.09	−0.01	0.04	0	0.11	0	0.11	−0.04	0.11	0
Total Variance	0.358129		0.358234		0.350256		0.319554		0.366478		0.301333	
Total SD	0.598438		0.598527		0.591824		0.565291		0.605374		0.548938	

PE, predicted error; TK, total keratometry from IOLMaster 700; TCP, total corneal power from Anterion; WTR, with-the-rule astigmatism; ATR, against-the-rule astigmatism; Act-pre, actual minus preoperative; x, x-PE component; y, y-PE component.

When subgroup analysis was performed, the WTR eyes had centroid PE of 0.17D@64° ± 0.60D for TK, aligned along the vertical axis, namely, the WTR prediction error; and 0.15D@31° ± 0.61D for TCP. In contrast, the ATR eyes had centroid value of 0.13D@17° ± 0.59D for TK and 0.15D@17° ± 0.55D for TCP, both were ATR prediction errors.

## 4 Discussion

Accurate astigmatism correction requires accurate measurement and calculation (18). At present, there are several instruments to measure total corneal astigmatism, which improves the accuracy of toric IOL calculation (10, 11). Both IOLMaster 700 and Anterion are based on swept-source optical coherence tomography. Previous studies have shown good repeatability and agreement of biometric measurements between them (19). Different from previous research, we evaluated the accuracy of TK from IOLMaster 700 and TCP from Anterion in astigmatism prediction based on Z CALC total keratometry mode. Strengths of the current study include the evaluation of TCA from two SS-OCT biometers (IOLMaster 700 and Anterion) in astigmatic prediction accuracy instead of PCA, the comparison of bivariate variances of two SS-OCT biometers, the inclusion of one eye per patient, and the use of confidence convex polygons instead of confidence ellipses on Double-angle plots.

No significant difference was found in the bivariate variances of preoperative corneal astigmatism between TK and TCP (5.1223 versus 5.5019, *P* = 0.081). The bivariate variances between preoperative corneal astigmatism and postoperative refractive astigmatism were compared for each method, and the difference reached statistical significance (5.1223 & 5.5019 versus 0.3899, both *P* < 0.001). Compared to the preoperative corneal astigmatism measured with TK and TCP, postoperative refractive astigmatism was significantly smaller, and the percentages of eyes within certain amounts of astigmatism magnitudes were significantly higher after surgery. The above outcomes indicated that each method significantly improved the postoperative refractive astigmatism.

The mean vector PEs were comparable between TK and TCP (0.48D versus 0.46D, *P* = 0.281). Compared with TK, the percentages of eyes with TCP increased by 12.7 and 5.4% with vector PEs of ≤ 0.25D and ≤ 0.5D, respectively. Although there was no statistical difference between the two methods, it did indicate that, if toric IOL calculation was performed using TCP from Anterion, 1 in 10 eyes may obtain more accurate refractive astigmatism results. The reasons for the difference could be as follows: the IOLMaster 700 uses a combination of SS-OCT and telecentric technology to directly measure the corneal curvature at a total of 18 points in the central 1.5, 2.5, and 3.5 mm rings (20). In comparison, the Anterion, based on SS-OCT technology, rapidly captures the corneal curvature of the central 3 mm ring of the cornea. A total of 65 radial B-scan images are obtained in a single scan, 256 A-scans per B-scan, which can theoretically calculate TCP more accurately (21).

Our results were compatible with previously published findings (10). Choi et al. (10) compared the astigmatic prediction accuracy of TCA from IOLMaster 700 and Pentacam when implanting Acrysof IQ Panoptix toric TFNT IOLs. In their study, the PE was ≤ 0.50 D for 45.5% (66/145) eyes when calculating based on TK, in contrast, 42.8% (62/145) eyes were ≤ 0.50 D when TCRP4 was used. For TK and TCRP4, the MAE were 0.60D and 0.61D, respectively (10). Our results are slightly higher than those of Choi et al. (10) possibly for the following three reasons: (1) The toric IOL calculators adopted in these studies were different: Z CALC 2.0 was used in current study, while Choi et al. used Alcon online calculator; (2) Different toric IOLs were implanted: The implanted IOL in this study was ZEISS 709M toric IOL, and Choi et al. inserted Acrysof IQ Panoptix toric TFNT IOL. It was showed that different designs of toric IOLs may result in different ELPs, which may affect the postoperative results (22). (3) Different biometers were utilized in the two studies.

In the current study, the x-PE component and y-PE component for TK and TCP were not normally distributed, and 95% confidence convex polygons were performed. No significant differences were found in x-PE component and y-PE component between TK and TCP. In subgroup analysis, the centroid PEs of TK and TCP in the WTR group were 0.17D@64° ± 0.60D and 0.15D@31° ± 0.61D, respectively, and those in the ATR group were 0.13D@17° ± 0.59D and 0.15D@17° ± 0.55D, respectively. This indicated that the IOLMaster 700 may have overestimated PCA in the WTR eyes and underestimated PCA in the ATR eyes, and the Anterion may have underestimated PCA in all eyes. Since the sample size in each group was not large enough and the SD values were large, subsequent studies need to include more eyes to draw a conclusion about whether these differences were in fact significant.

The limitation of our study is that the total corneal curvature measured preoperatively was used in the toric IOL calculation, and the SIA may have an impact on the analysis. The SIA used in the study is the average personalized SIA previously calculated for the same surgeon. Clinical practice can be simulated by using preoperative parameters and a personalized SIA. In addition, the sample size of the study was relatively small, only 20 WTR eyes were included, and oblique astigmatism was not included. Therefore, a larger population study is needed to evaluate the role of TCA in toric IOL calculation. Furthermore, further studies are needed to be extrapolated to other similar devices.

## 5 Conclusion

This study showed that, compared with TK from the IOLMaster 700, TCP from the Anterion achieved comparable accuracy in residual astigmatism prediction and resulted in 12.7 and 5.4% more eyes with vector PEs of ≤ 0.25D and ≤ 0.5D, respectively. This demonstrates that Anterion, as a new SS-OCT biometer, may provide a new choice for astigmatism prediction.

## Data availability statement

The raw data supporting the conclusions of this article will be made available by the authors, without undue reservation.

## Ethics statement

The studies involving humans were approved by the Shanxi Eye Hospital Institutional Review Board (No. SXYYLL-20210107). The studies were conducted in accordance with the local legislation and institutional requirements. The participants provided their written informed consent to participate in this study.

## Author contributions

FC: Conceptualization, Data curation, Funding acquisition, Writing – original draft, Writing – review & editing. JM: Conceptualization, Software, Writing – review & editing, Methodology. XW: Conceptualization, Funding acquisition, Writing – original draft, Writing – review & editing. JiL: Formal analysis, Investigation, Validation, Writing – review & editing, Resources. LJ: Formal analysis, Validation, Visualization, Writing – review & editing, Investigation. WW: Data curation, Validation, Writing – review & editing, Formal analysis, Investigation. JuL: Conceptualization, Project administration, Supervision, Writing – original draft, Writing – review & editing, Methodology.

## Footnotes

1
https://www.r-project.org/
